# The Role of the Selective Adaptor p62 and Ubiquitin-Like Proteins in Autophagy

**DOI:** 10.1155/2014/832704

**Published:** 2014-06-12

**Authors:** Mónika Lippai, Péter Lőw

**Affiliations:** Department of Anatomy, Cell and Developmental Biology, Eötvös Loránd University, Pázmány Péter sétány 1/C., Budapest 1117, Hungary

## Abstract

The ubiquitin-proteasome system and autophagy were long viewed as independent, parallel degradation systems with no point of intersection. By now we know that these degradation pathways share certain substrates and regulatory molecules and show coordinated and compensatory function. Two ubiquitin-like protein conjugation pathways were discovered that are required for autophagosome biogenesis: the Atg12-Atg5-Atg16 and Atg8 systems. Autophagy has been considered to be essentially a nonselective process, but it turned out to be at least partially selective. Selective substrates of autophagy include damaged mitochondria, intracellular pathogens, and even a subset of cytosolic proteins with the help of ubiquitin-binding autophagic adaptors, such as p62/SQSTM1, NBR1, NDP52, and Optineurin. These proteins selectively recognize autophagic cargo and mediate its engulfment into autophagosomes by binding to the small ubiquitin-like modifiers that belong to the Atg8/LC3 family.

## 1. Introduction

Two major pathways accomplish regulated protein catabolism in eukaryotic cells: the ubiquitin-proteasome system (UPS) and the autophagy-lysosomal system. The UPS serves as the primary route of degradation for thousands of short-lived proteins and many regulatory proteins and contributes to the degradation of defective proteins [1]. Autophagy, by contrast, is primarily responsible for degrading long-lived proteins and maintaining amino acid pools during stress conditions, such as in chronic starvation [2]. The critical factors that direct a specific substrate to one degradation route or the other are incompletely understood. Protein degradations performed by the UPS and autophagy were regarded for a long time as complementary but separate mechanisms [3]. However, on the basis of recent studies, there are overlaps between them. The way of degradation of a misfolded, redundant, or unneeded protein may be often governed by the momentary activity or capacity of these systems or, in some cases, determined by strict regulation. Moreover, the two pathways use common adaptors capable of directing ubiquitinylated target proteins to both.

## 2. Ubiquitin-Proteasome System

**Figure 1:**
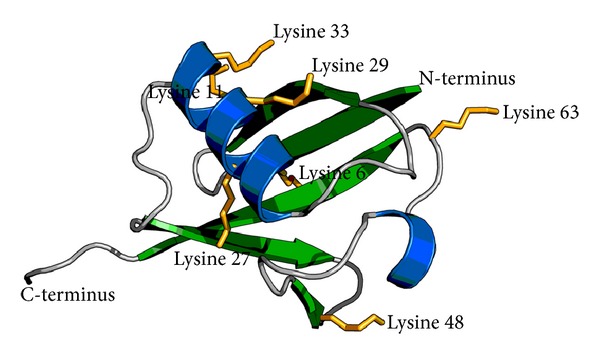
Ribbon model of ubiquitin exposing all the seven lysine side chains possibly involved in polyubiquitinylation reactions.

The ubiquitin-proteasome pathway plays a crucial role in governing many basic cellular processes, such as normal protein turnover, protein quality control by degrading misfolded and damaged proteins, signal transduction, metabolism, cell death, immune responses, and cell cycle control [4]. Ubiquitin is a small, globular protein containing 76 amino acid residues (Figure 1). There are only three amino-acid changes from yeast to human, so ubiquitin is highly conserved within eukaryotes. Ubiquitinylation, the covalent conjugation of ubiquitin to other proteins, is a special posttranslational modification, which may either serve as an essential degradation signal for proteins or it may alter their localisation, function, or activity.

**Figure 2:**
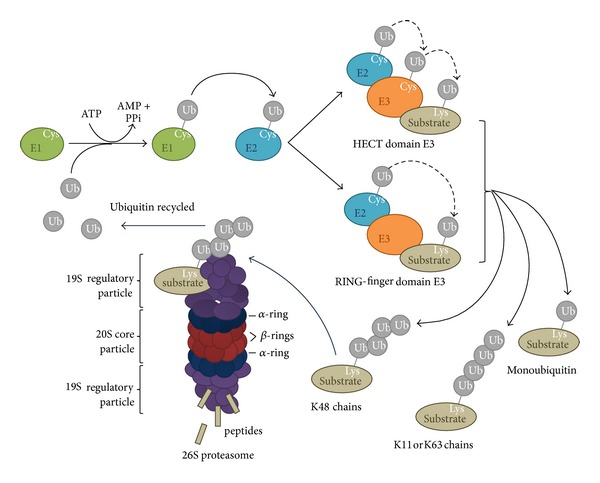
The ubiquitin-proteasome system. An enzyme cascade organizes the attachment of mono- or polyubiquitin to the substrates. Ubiquitin (Ub) is first activated in an ATP-consuming reaction by E1 (Ub-activating enzyme), to which it becomes attached by a high-energy thiolester bond. Then, the activated Ub is shifted to the active Cys residue of E2 (ubiquitin-conjugating enzyme). E2 catalyzes the transfer of ubiquitin to the substrate protein with the help of E3 (ubiquitin ligase). There are two major classes of E3 enzymes, characterized by the HECT domain or the RING-finger domain. In case of the HECT E3 enzymes, the activated Ub is transferred first to an active Cys residue in the HECT domain before it is finally moved to the substrate. RING-finger domain E3 enzymes bind to both the E2 enzyme and the substrate and catalyze the transfer of Ub directly from the E2 enzyme to the substrate. A polyubiquitin chain linked through Lys 48 is the signal for the proteasome to degrade the substrate. The 26S proteasome consists of the catalytic 20S core particle; a barrel of four stacked rings: two outer *α*-rings (blue) and two inner *β*-rings (red); and the 19S regulatory particle. The polyubiquitin chain is recognized by the regulatory particle, which then binds, unfolds, and translocates the polypeptide into the catalytic core. The substrate is hydrolyzed by the enzymatically active *β*-subunits inside the core particle producing short peptides. Ubiquitin is recycled in the process [102, 103].

Before being covalently attached to other proteins, free ubiquitin is activated in an ATP-dependent manner with the formation of a thiolester linkage between a ubiquitin-activating enzyme (E1) and the carboxyl terminus of ubiquitin. Then, it is transferred to a ubiquitin-conjugating enzyme (E2). Finally, E2 associates with ubiquitin-ligases (E3s) which specifically bind the target substrate and attach ubiquitin through its carboxyl terminal glycine to the *ε*-amino group of a lysine residue in the target protein (Figure 2). The exact details of ubiquitinylation biochemistry are determined by the type of E3 enzyme involved. E3s can be grouped into two major classes: HECT (homologous to E6-AP carboxyl-terminus) domain E3s and RING-finger (really interesting new gene) domain E3s [5]. The identification of E6-AP as the E3 responsible for the human papilloma virus E6-dependent ubiquitinylation of p53 led to the discovery of the HECT domain enzymes [6]. HECT domain is a conserved C-terminus of the molecule, which is about 350 amino acids long. HECT domain E3s form thiolester intermediates with ubiquitin through a conserved cysteine residue, like in case of E1 and E2 enzymes. By contrast, RING-finger E3s do not generate a thiolester intermediate but just simply act as a scaffold to hold a ubiquitin-E2 intermediate close to a substrate and catalyze ubiquitin transfer [7] (Figure 2).

The high specificity of the UPS system is tightly associated with the E3 enzymes, as they determine which substrate should be ubiquitinylated and hence usually degraded. Whether the attached ubiquitin is a modification signal or a sign for degradation depends on how it is linked to its substrates: conjugation of a single ubiquitin monomer (monoubiquitinylation) or sequential conjugation of several ubiquitin moieties (polyubiquitinylation) of variable length.

The ubiquitin chain could be lengthened by the E2 and E3, sometimes with the help of an accessory factor (E4). The carboxyl terminal glycine of the more distal ubiquitin molecule is bound to the previous ubiquitin molecule through an isopeptide bond with an *ε*-amino group of a lysine [8]. If the series of ubiquitin moieties is extended to at least four units, then it is sufficient to allow the ubiquitylated target protein to be recognized and degraded by the 26S proteasome [9].

The 26S proteasome is a 2.5 MDa multicatalytic multisubunit protease, which is made up of two subcomplexes: a barrel-shaped core particle (CP: also known as the 20S proteasome) and one or two 19S regulatory particle(s) (RP) on one or both ends of the core particle [10–12]. The 19S RP serves to recognize ubiquitinylated substrate proteins and plays a role in their unfolding and translocation into the interior of the 20S CP (Figure 2).

The 20S CP contains two outer *α*-rings and two inner *β*-rings, each of which is made up of seven structurally similar *α* and *β* subunits, respectively. The rings form an *α*_1–7_ *β*_1–7_ *β*_1–7_ *α*_1–7_ structure creating three continuous chambers inside the particle. Only three of the *β*-type subunits (*β*1, *β*2, and *β*5) in each inner ring are catalytically active. They have threonine residues at their N-termini and show N-terminal nucleophile hydrolase activity. Such a “self-compartmentalized” structure keeps the proteolytic active sites separated in the central chamber and allows regulated substrate degradation only. The proteasome is a multicatalytic protease because the *β*1, *β*2, and *β*5 subunits are associated with caspase-like, trypsin-like, and chymotrypsin-like activities, respectively, which are able to cleave amide bonds at the C-terminal side of acidic, basic, and hydrophobic amino-acid residues, respectively.

The ubiquitin chains are called K6, K11, K27, K29, K33, K48, or K63 chains depending on which of the seven lysine (K) residues is involved in linkage of monomers in the polyubiquitin polymer (Figures 1 and 2). K48 ubiquitin chain was first identified as the signal to target proteins for proteasomal degradation. In contrast, K11 or K63 chains or single ubiquitin moieties (monoubiquitinylation) were thought to signal mainly for nonproteolytic functions [13]. These chain types are involved in controlling several processes such as gene transcription, DNA repair, cell cycle progression, apoptosis, and receptor endocytosis [14]. However, recent reports have demonstrated that all types of ubiquitin chains as well as monoubiquitinylation can target substrates for degradation via autophagy [15].

## 3. Ubiquitin-Like Proteins

**Figure 3:**
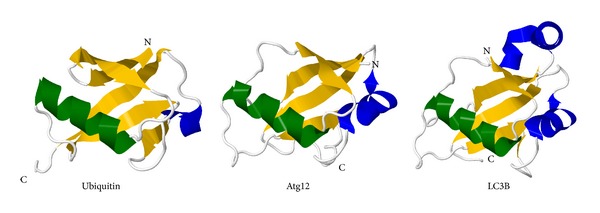
Structures of ubiquitin and the ubiquitin-like proteins (Ubls) Atg12 and LC3B, shown as ribbon diagrams generated by Jmol 13.0 [104] upon the structural data deposited in PDB. The characteristic Ubl *β*-grasp fold: a *β*-sheet with four antiparallel *β*-strands (yellow) and a helical segment (green) is well observable. Other helical structures are blue (Protein Data Bank (PDB) accession codes: 1UBQ [105], 4GDK [106], and 1UGM [107], resp.).

There are more and more ubiquitin-like proteins (Ubls) identified and characterized. They resemble ubiquitin, as for all Ubls whose covalent attachment to other biomolecules has been experimentally demonstrated, the C-terminal residue is a glycine, and the carboxyl group of this glycine is the site of attachment to substrates [16]. On substrate proteins lysine side chains are the target sites so the Ubl and substrate are connected with an amide (or isopeptide) bond. Ubls also share a similar structural motif, the *β*-grasp fold, which contains a *β*-sheet with four antiparallel *β*-strands and a helical segment (Figure 3).

## 4. Autophagy

**Figure 4:**
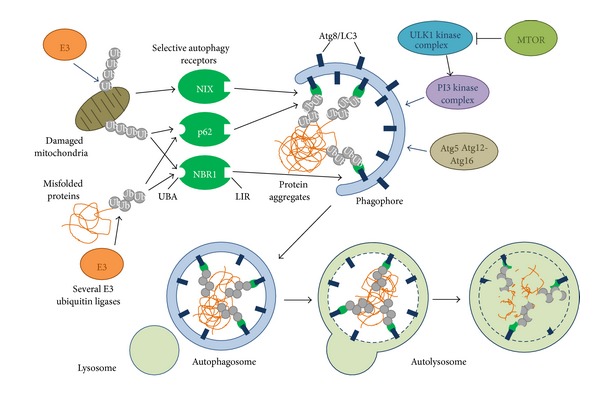
The process of autophagy. Initiation of autophagy is controlled by the ULK1 complex, followed by activation of the PI3-kinase complex leading to nucleation of the phagophore. Vesicle expansion is governed by two ubiquitin-like conjugation systems: the Atg5-Atg12- Atg16 and Atg8/LC3 pathways. Finally, autophagosomes fuse with lysosomes forming autolysosomes, where breakdown of the autophagic cargo takes place. Selective autophagy can distinguish and direct specific cargos to the lysosome. Autophagy receptors contain a short LIR (LC3-interacting region) sequence responsible for Atg8/LC3 binding. Recognition of ubiquitinylated proteins is mediated by interacting with ubiquitin noncovalently, via an ubiquitin-binding domain (UBA). NIX acts as a mitophagy receptor; it has a LIR motif but lacks an UBA domain and is localized within the mitochondrial outer membrane; this is why ubiquitinylation is not required for NIX-dependent delivery of damaged mitochondria to autophagosomes.

Autophagy is another degradative pathway that occurs in all eukaryotic cells. It is the main system for the degradation of bulk cytoplasmic components in the cell, and it is induced by nutrient starvation for example. Autophagy is crucial for homeostasis in the cell, as it recycles proteins and organelles. In addition, autophagy plays a critical role in cytoprotection by preventing the accumulation of toxic proteins and acting in various aspects of immunity, including the elimination of invading microbes and its participation in antigen presentation. Macroautophagy is the best characterized type of autophagy. In this case the cell forms a double-membrane sequestering compartment called the phagophore, which develops into an autophagosome. After fusion with lysosomes, the content of the resulting autolysosome is degraded and the newly generated monomers are released back into the cytosol for reuse [2, 17] (Figure 4).

There are 38 known autophagy-related (Atg) genes regulating the steps of autophagosome formation and breakdown. These were identified in yeast genetic screens but they are evolutionarily well conserved also in plants and animals, including* Drosophila* and mammalian cells [18, 19]. Initiation of autophagy is controlled by the Atg1/ULK complex, consisting of Atg1, Atg13, Atg17, Atg29, and Atg31 in yeast and ULK1/2, mAtg13, FIP200, and Atg101 in mammals. The ULK1/2, mAtg13, and FIP200 proteins form a complex independently of nutrient supply. MTORC1 (mechanistic target of rapamycin complex 1) phosphorylates and inhibits ULK1/2 and mAtg13 in nutrient-rich conditions, disrupting the contact between ULK1 and AMPK, an energy sensor kinase with activating effect on ULK1. On the contrary, MTOR is released from its complex under starvation, resulting in activation of ULK1/2 (Figure 4), which, in turn, phosphorylates and activates mAtg13 and FIP200 [20].

The transmembrane protein Atg9 and regulators of its trafficking (Atg2 and Atg18) play a role in membrane delivery to the expanding phagophore after the assembly of the Atg1 complex at the single phagophore assembly site (PAS), which is marked by the selective cargo proaminopeptidase I aggregate in yeast. Nucleation of the phagophore at the PAS is controlled by the phosphatidylinositol-3-kinase (PI3 K) complex (Vps34/hVPS34, Vps15/hVPS15, Vps30/Atg6/Beclin 1, and Atg14/ATG14L). Finally, there are two Ubl conjugation systems: the Atg12 (Atg5, Atg7, Atg10, Atg12, and Atg16) and Atg8 (Atg3, Atg4, Atg7, and Atg8) pathways which are responsible for vesicle expansion [18, 21] (Figure 4).

Autophagosomes undergo a maturation process in animal cells, which involves the recruitment of the SNARE protein syntaxin 17 [22–24]. Interaction of syntaxin 17 with the HOPS (homotypic fusion and vacuole protein sorting) tethering complex promotes the fusion of autophagosomes with lysosomes, where breakdown of autophagic cargo takes place [25, 26] (Figure 4).

Macroautophagy has long been considered as a nonselective process responsible for bulk degradation of cytoplasmic components. The autophagy pathway appeared during evolution as an adaptation mechanism of the eukaryotic cell to starvation, allowing mobilization of nutrients in the cell by forfeit materials of the cytosol. Additionally, it became indispensable for specific degradation of unnecessary or toxic structures: proteins, organelles, and intracellular pathogens [27]. In contrast to the bulk autophagy, which ensures the more or less random sequestration of cytosol, selective autophagy operates under nutrient-rich conditions as well and is characterized by the presence of specialized autophagosomes. These autophagosomes lock up substrates in an exclusive way, which means that other parts of the cytoplasm are largely absent from them [18, 28, 29] (Figure 4).

### 4.1. Atg12 and Atg8

Autophagy requires the Ubls Atg12 and Atg8/LC3 (Figures 3 and 4). Atg12, which is 2.5 times larger than ubiquitin, was the first Ubl identified as a core autophagy protein [30]. It is synthesized in an active form that does not require proteolytic maturation. The C-terminal glycine of Atg12 is first activated by the E1 enzyme Atg7, and is then transferred to an E2 enzyme, Atg10, before finally forming a conjugate with Atg5 [30]. This Atg12-Atg5 conjugate is essential for autophagy. This system is well conserved in mammals; there is only one orthologue for each of the components of the Atg12 system in mice and humans [21].

Atg8, the other Ubl regulator of autophagy, is expressed with a C-terminal arginine residue in yeast, which is removed by the cysteine protease Atg4 leaving a glycine residue at the C-terminus [31]. Biochemical studies revealed the existence of another ubiquitinylation-like conjugation system [32]. The C-terminal glycine residue of Atg8 is activated by the same E1-like enzyme, Atg7, as in case of Atg12. Then Atg3, an E2-like enzyme, together with an Atg12-5-16 complex catalyzes the transfer of the activated Atg8 to phosphatidylethanolamine, the target lipid substrate. This way Atg8 becomes tightly membrane associated. Atg8 therefore can be utilized as a marker of the autophagosomal membrane and a key molecule during autophagosome formation (Figures 3 and 4). The conjugation of Atg8 to and its removal from phosphatidylethanolamine are essential for autophagy. There are three families of Atg8 homologues in mice and humans called LC3s, GABARAPs, and GABARAP-like proteins.

### 4.2. Selective Autophagy and Its Specific Adaptors

In the last decade, emerging evidence revealed that autophagy can distinguish and direct specific cargos to the lysosome. Different terms were coined to distinguish between different targets. The most investigated processes are mitophagy: the selective removal of defective or excess mitochondria [33]; aggrephagy: the disposal of aberrant, misfolded protein aggregates [34]; xenophagy: the selective autophagy of pathogenic intracellular bacteria, protozoa, or viruses [35, 36], and pexophagy: peroxisome autophagy first described in detail during peroxisome degradation in methylotrophic yeast species but also responsible for the destruction of 70–80% of the peroxisomal mass in mammalian cells [37]. The selective nature of autophagy is ensured mainly by specific adaptors, but direct interactions between the target molecule and the core autophagy machinery are also observed.

A molecule convenient to link a process with its substrate needs to carry at least two distinct functional domains: one that recognizes the target and another that transports it to the site of operation. How does it work in the case of selective autophagy? The best known mechanism to solve the problem of distinction between the different cytoplasmic components deemed for engulfment is to bring properly marked cargos to the inner surface of the growing phagophore. Accordingly, the precise delivery is generally ensured by interaction of the adaptor both with the membrane-anchored form of Atg8/LC3 and the main targets that are usually polyubiquitinylated (Figure 4).

The first clues for the role of protein ubiquitinylation as a signal for selective autophagy came from Atg knockout mice and some* Drosophila *experiments. They showed that the loss of basal autophagy in the brain resulted in large-scale accumulation of ubiquitinylated proteins [38–40].

**Figure 5:**
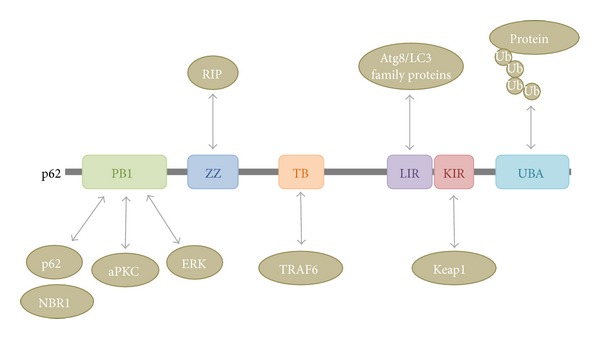
Domain structure of p62 and its interacting partners. There are six main domains/motifs in the p62 protein, necessary for its interaction with the autophagic machinery and with signaling pathways. The N-terminal Phox and Bem1 (PB1, 21-103 aa) domain is involved in the self-oligomerization of p62 or in heterodimerization with NBR1, a protein similar to p62. The PB1 domain is also responsible for the binding to atypical PKC (aPKC) or to ERK1. The central zinc finger ZZ domain (128-163 aa) and the TRAF6-binding domain (TB, 225-250 aa) interact with the RIP and TRAF6 proteins, respectively, to regulate the NF-*κ*B pathway. Through the LC3-interacting region (LIR, 321-345 aa) and the C-terminal ubiquitin-associated domain (UBA, 386-440 aa), p62 links the autophagic machinery to ubiquitinylated protein substrates to promote the selective degradation of these molecules. Finally, the Keap-interacting region (KIR, 346-359 aa) binds Keap1 leading to stabilization and nuclear translocation of the transcription factor Nrf2, engaged in the control of ROS level.

Recognition of ubiquitinylated proteins during autophagy is mediated by ubiquitin receptors interacting with ubiquitin noncovalently, via their ubiquitin-binding domains. p62/SQSMT1 (hereafter p62), the first protein reported to have such an adaptor function [41], was originally discovered as a scaffold in signaling pathways regulating cell growth and proliferation; however, it was also detected in ubiquitinylated protein aggregates [42] (Figure 4). p62 possesses a C-terminal ubiquitin-binding domain (UBA) [43] and a short LIR (LC3-interacting region) sequence responsible for LC3 interaction [41]. In addition, it has a PB1 domain promoting self-aggregation and association with other adaptors such as NBR1, neighbour of BRCA1 gene 1 [15] (Figure 5). Knockout studies in mice and* Drosophila* revealed that p62 is required for the aggregation of ubiquitinylated proteins and thus plays essential roles for their autophagic clearance [44, 45]. The levels of p62 usually inversely correlate with autophagic degradation, as the loss of Atg genes or factors required for the fusion of autophagosomes with lysosomes all result in a marked increase of p62-positive aggregates [46, 47]. p62 can also deliver ubiquitinylated cargos to the proteasome, although they are mainly degraded by autophagy [48, 49].

Another adaptor used in selective autophagy is the above-mentioned* NBR1*, which, via its own PB1 domain, is able to interact with p62, and through its own UBA domain and LIR it can participate in the recruitment and autophagosomal degradation of ubiquitinylated proteins [50]. In plants, a functional hybrid homologue of p62 and NBR1 (NBR1 in Arabidopsis, Joka2 in tobacco) plays an important role in the disposal of polyubiquitinylated proteins accumulated under abiotic stress conditions [51, 52].

*Optineurin* and* NDP52* have been recently described as xenophagy receptors, utilizing the autophagic machinery for restriction of ubiquitinylated intracellular pathogens [53]. Both of them also participate in the clearance of protein aggregates [54, 55] and are required for the regulation of NF-*κ*B signaling [56, 57].

While these receptors all mediate degradation of ubiquitinylated cargos, there are other more specific adaptors acting on removal of damaged or surplus mitochondria (e.g., Atg32 in yeast and NIX in mammals) or peroxisomes (such as Atg30 and Atg36). They recognize particular binding partners on the surface of their target organelle and, through their LIR sequence, ensure their delivery to the maturing autophagosome [58, 59]. It is worth noting that additional autophagic adaptors may be identified by software prediction of LIR sequences in suspected protein candidates [60] (see a recent review for more details on the structural basis of how the Atg8/LC3 and Atg12 Ubls interact with specific autophagy adaptors [21]).

#### 4.2.1. Role of p62 in Autophagosome Formation

As individual p62-ubiquitin interactions are rather weak, the starting point of the polyubiquitinylated aggregate formation is presumably the p62 self-oligomerization via its PB1 domain [61]. However, the original “simple” concept of delivery through bridging the polyubiquitin side chain on the cargo and the Atg8/LC3 decoration on the phagophore surface by p62 is now changing. In fact, these aggregates containing p62 and ubiquitinylated proteins may even serve as a nucleating scaffold for autophagosome biogenesis, potentially by binding multiple Atg proteins [61–63].

Moreover, it was recently reported that phagophores may preferentially form at p62 aggregates near lysosomes in* Drosophila* cells, which is very similar to the location of PAS near the vacuole/lysosome in yeast [64, 65]. It is worth noting that p62 also associates with MTORC1 [66]. MTORC1 is active when bound to lysosomes and promotes cell growth and inhibits autophagy by phosphorylating Atg1 (ULK1/2) [67–69]. These data suggest the direct assembly of early autophagic structures on the surface of protein aggregates, which may be mediated by interactions between p62 and upstream Atg proteins. Later on, Atg8/LC3 will be recruited to the forming phagophore, and the growing double membrane will enclose the p62-containing aggregate due to interactions between p62, Atg8/LC3, and other Atg proteins [70, 71].

#### 4.2.2. p62 in Autophagy Regulation

The role of p62 in the regulation of autophagy is controversial. It was suggested to promote MTORC1 activation by contributing to its translocation to the lysosomal surface. Therefore, p62 reduction, similarly to MTORC1 inactivation, may activate autophagy [72]. However, in HEK293 and HeLa cells p62 was suggested to liberate Beclin1 (an Atg6 homologue) by disrupting the association of Bcl-2 and Beclin1, and thus p62 may positively regulate the induction of bulk autophagy [73]. In addition, p62 interacts with and regulates the deacetylase activity of HDAC6, a known modifier of F-actin network involved in selective autophagy [74]. In carcinoma cells, while p62 silencing suppressed cell proliferation and induced autophagy, abnormal autophagosomes were found and p62 inhibition finally resulted in autophagic cell death [75]. We have recently found that p62 is not required for proteasome inhibition-induced autophagy in* Drosophila* fat body cells [76]. Thus, the role of p62 in autophagy induction appears to be complex and probably context-dependent.

As p62 can shuttle between the nucleus and the cytoplasm (in the nucleus it is thought to recruit proteasomes to nuclear polyubiquitinylated protein aggregates), it can even export ubiquitinylated substrates from the nucleus into the cytosol, where autophagy offers a more robust degradative capacity [77].

#### 4.2.3. Cytoplasmic p62 Level as an Autophagy Indicator

Since p62 itself is removed from the cytoplasm mainly by autophagy, its amount is generally considered to inversely correlate with autophagic activity [46, 47]. Accumulation of p62-positive inclusions during immunocytochemistry or elevated p62 levels on Western blots are frequently used as signs of autophagy impairment. In some cases, transgenic p62 reporter systems are also used to monitor the rate of autophagic degradation, although their use requires caution as overexpressed p62 tends to self-aggregate and may no longer indicate autophagy activity [78]. In addition, long term starvation may positively influence the amount of p62 in certain mammalian cell types, via both its transcriptional upregulation and promoting* de novo* p62 protein synthesis by providing autophagy-derived amino acids [49].

## 5. Interplay between p62 and Signaling Pathways

p62 was originally described as a scaffold protein ensuring the formation of signaling hubs, since, through different binding domains, it can establish interactions with many types of enzymes. As a consequence, it is able to integrate signaling routes involving particular kinases and ubiquitin-mediated pathways (Figure 5). This way, p62 regulates inflammatory processes in TNF*α*-activated cells. The complex including the RIP kinase, atypical PKCs and TRAF6, and a K63 ubiquitin ligase (interactions formed through the ZZ, PB1, and TB domain of p62, resp.) plays a critical role in the phosphorylation of IKK*β* leading to activation of the NF-*κ*B transcription factor [79]. Enhanced p62 level (under inflammatory conditions induced by impaired proteasomal degradation) was demonstrated to contribute to elevated IL-1*β* production: p62 was found to bind the JNK and ERK kinases, hence further increasing NF-*κ*B activation and, as a consequence, pro-IL-1*β* expression. In addition, p62 accumulation was found to promote caspase-1 activation in inflammasomes, which is required for IL-1*β* proteolytic processing [80]. Interestingly, an opposite effect of p62 is suggested in Legionella-infected p62-deficient mice that showed more severe pulmonary inflammation than control animals, because the production and secretion of IL-1*β* was significantly enhanced due to elevated caspase-1 activity in their macrophages [81].

p62, likewise in association with TRAF6 and aPKCs, is needed for the NF-*κ*B-mediated neuronal survival and differentiation in response to NGF [82] and also for osteoclastogenesis [83]. p62 mutations are among the genetic alterations that play a role in Paget disease of bone, where osteoclasts are overactive because of disturbed NF-*κ*B signalization [84]. The p62-NF-*κ*B connection has a role in tumorigenesis as well, since p62 is necessary to NF-*κ*B-dependent survival in Ras-transformed cells [85].

The autophagy adaptor function of p62 also has an impact on the NF-*κ*B signaling pathway. In human monocytes, high level of inflammation due to autophagy impairment is associated with p62 accumulation and the consequent overactivation of the NF-*κ*B pathway [86]. In accordance with the positive role of p62 in caspase-1 activation [80], a previous study demonstrated that stimulated autophagy, by enhanced degradation of p62, also eliminates activated inflammasomes and reduces inflammation, while blocking autophagy has an opposite effect [87]. In addition, NF-*κ*B signalization may be regulated directly by the rate of NF-*κ*B removal. Targeted degradation of the p62-NF-*κ*B p65 subunit complex by p62-mediated selective autophagy may play a key role in bone marrow derived macrophage differentiation [88].

The important role of p62 in innate immunity does not only rely on regulation of immune signaling responses. As an autophagy adaptor, p62 takes part in the elimination of ubiquitinylated intracellular pathogens; some infecting agents even target this step to escape from the defensive system of the cell. The coxsackievirus B3, through the activity of one of its proteases, cleaves p62 which results in impairment of selective autophagy and host defense [89]. Moreover, selective autophagy induced by pathogen-specific TLR4 activation requires transcriptional upregulation of p62 [90]. Interestingly, p62 also participates in the synthesis of neoantimicrobial peptides, by bringing inactive precursors such as Fau to autophagic degradation, where they are processed to active fragments [91].

p62 is also involved in the regulation of apoptosis. p62-mediated aggregation is needed for the activation of polyubiquitinated caspase-8 [92]. It was shown recently that caspase-8 colocalizes not only with p62, but also with Atg8/LC3 and Atg5, and its full self-processing requires the autophagosomal membrane as a platform for the assembly of the death-inducing signaling complex [93]. On the other hand, failure of autophagy may contribute to enhanced apoptosis because of impaired degradation of p62-complexed apoptosis proteins, as found in T-cells [94], while in autophagy-inhibited cancer cells, caspase-8 dependent cell death was mainly associated with the concomitantly elevated p62 level [95].

Another well-known signaling pathway influenced by p62 is the oxidative stress response, which is regulated by the Keap1-Nrf2 system. Through its KIR motif (Figure 5), p62 is able to bind to Keap1, a Cullin3-ubiquitin E3 ligase complex adaptor protein. In turn, Keap1-promoted polyubiquitinylation and subsequent proteasomal degradation of the transcription factor Nrf2 are inhibited. As a consequence, the expression of cytoprotective, antioxidant Nrf2 target genes is increased [96, 97]. Moreover, the p62 gene itself is a target for Nrf2; thus, the appropriate oxidative stress response is supported by a positive feedback regulation between p62 and Nrf2 [98]. Autophagy has a strong impact on Nrf2 activation, since p62 not only disrupts Keap1-Nrf2 interaction but also removes Keap1 from the cytosol via selective autophagy [99]. The well-known antioxidant effect of sestrins is, at least partly, due to their influence on the p62-dependent autophagic degradation of Keap1 [100]. In case of autophagy impairment, accumulation of p62 and the subsequent overactivation of Nrf2 may contribute to development of liver carcinomas [96]. Interestingly, in these cancer cells, phosphorylation of p62 by the MTORC1 complex increases its affinity for Keap1, so MTORC1 activity further enhances stabilization of Nrf2 and the transcription of its target genes [101].

## 6. Conclusions

- Ubiquitin and ubiquitin-like proteins (Ubl) share functional similarity. The different Ubls are activated and conjugated to substrates by similar biochemical mechanisms.
- Ubiquitinylation is frequently needed for substrate recognition and renders selectivity to autophagy in eukaryotes.
- The connection between ubiquitinylation and autophagy is provided by autophagic adaptor proteins (or autophagy receptors), which bind both ubiquitin and autophagy specific Ubl modifiers like Atg8/LC3 family proteins.
- Atg8/LC3 is required for the biogenesis of autophagosomal membrane and also mediates selective autophagy via the recruitment of LIR-containing autophagy receptors that recognize and select cargo.
- Autophagy receptors such as p62 regulate the selective autophagosomal degradation of large protein aggregates, mitochondria, and bacterial pathogens.
- p62 may play an important role also as a regulator of autophagy; moreover, it may even be involved in the formation of the autophagosome.
- As a scaffold protein, p62 operates in signaling pathways which, through the link provided by p62, can also be regulated by selective autophagy.
